# Antibiotic Use in Thailand: Quantifying Impact on Blood Culture Yield and Estimates of Pneumococcal Bacteremia Incidence

**DOI:** 10.4269/ajtmh.2010.09-0584

**Published:** 2010-08-05

**Authors:** Julia Rhodes, Joseph A. Hyder, Leonard F. Peruski, Cindy Fisher, Possawat Jorakate, Anek Kaewpan, Surang Dejsirilert, Somsak Thamthitiwat, Sonja J. Olsen, Scott F. Dowell, Somrak Chantra, Kittisak Tanwisaid, Susan A. Maloney, Henry C. Baggett

**Affiliations:** International Emerging Infections Program, Thailand Ministry of Public Health – United States Centers for Disease Control and Prevention Collaboration, Nonthaburi, Thailand; Department of Anesthesiology, Mayo Clinic, Rochester, Minnesota; Johns Hopkins University School of Medicine, Baltimore, Maryland; National Institute of Health, Ministry of Public Health, Nonthaburi, Thailand; Centers for Disease Control and Prevention, Atlanta, Georgia; Crown Prince Hospital, Thailand Ministry of Public Health, Sa Kaeo, Thailand; Nakhon Phanom Hospital, Thailand Ministry of Public Health, Nakhon Phanom, Thailand

## Abstract

No studies have quantified the impact of pre-culture antibiotic use on the recovery of individual blood-borne pathogens or on population-level incidence estimates for *Streptococcus pneumoniae*. We conducted bloodstream infection surveillance in Thailand during November 2005–June 2008. Pre-culture antibiotic use was assessed by reported use and by serum antimicrobial activity. Of 35,639 patient blood cultures, 27% had reported pre-culture antibiotic use and 24% (of 24,538 tested) had serum antimicrobial activity. Pathogen isolation was half as common in patients with versus without antibiotic use; *S. pneumoniae* isolation was 4- to 9-fold less common (0.09% versus 0.37% by reported antibiotic use; 0.05% versus 0.45% by serum antimicrobial activity, *P* < 0.01). Pre-culture antibiotic use by serum antimicrobial activity reduced pneumococcal bacteremia incidence by 32% overall and 39% in children < 5 years of age. Our findings highlight the limitations of culture-based detection methods to estimate invasive pneumococcal disease incidence in settings where pre-culture antibiotic use is common.

## Introduction

Antibiotic use is common among pediatric and adult patients presenting in both inpatient and outpatient settings.^1^ The scope and magnitude of antibiotic use is not well defined, particularly in low and middle income nations where antibiotics are often available without prescription. Antibiotic pre-treatment decreases the already low sensitivity of blood culture for detecting bacterial causes of pneumonia and sepsis in adults^2^ and children.^3^,^4^ However, no studies have quantified the impact of antibiotic use on the recovery of specific bacterial pathogens and more importantly its impact on population-level incidence estimates for individual diseases such as those caused by *Streptococcus pneumoniae*.

The World Health Organization (WHO) recommends that all countries prioritize the inclusion of the heptavalent pneumococcal conjugate vaccine (PCV7) in national vaccine programs.^5^ Countries considering vaccine introduction often use invasive pneumococcal disease (IPD) incidence estimates as the primary indicator of disease burden, because accurate measures of other forms of pneumococcal disease (e.g., pneumonia) are difficult to obtain outside of vaccine probe studies. Accurate IPD estimates are therefore of critical public health importance and the impact of antibiotic use on these estimates is unknown. Using active, population-based surveillance from two provinces in rural Thailand, we measured the prevalence of antibiotic use before blood culture and its impact on *S. pneumoniae* isolation and on estimates of the incidence of pneumococcal bacteremia requiring hospitalization.

## Materials and Methods

### Setting.

The International Emerging Infections Program (IEIP), a collaboration between the Thailand Ministry of Public Health and the U.S. Centers for Disease Control and Prevention (CDC), has conducted an active, population-based surveillance for pneumonia requiring hospitalization since 2002 in two rural provinces: Nakhon Phanom (population 734,000), on the border of Laos in northeastern Thailand, and Sa Kaeo (population 526,000), adjacent to Cambodia in eastern Thailand.^6^ Since May 2005, IEIP has also conducted enhanced surveillance for bacteremia requiring hospitalization in these same provinces.^7^ Surveillance is conducted at all hospitals in the two provinces: 16 community (local) and 2 military hospitals (10 to 140 inpatient beds) and both provincial (referral) hospitals (225 and 327 inpatient beds).

Clinicians requested blood cultures from hospitalized patients as clinically indicated. Cultures were actively encouraged in all patients admitted with suspected pneumonia and patients < 5 years of age with suspected sepsis. To minimize potential confounding from patients with multiple blood cultures, data were limited to cultures performed at least 7 days apart (i.e., repeat cultures within 7 days were excluded); cultures obtained from the same patient ≥ 7 days apart were treated as unique patient encounters. Detailed clinical and demographic information were available for the subset of patients with blood cultures who were also captured in the pneumonia surveillance system,^7^ whereas more limited data were available for other patients with blood cultures.

### Antibiotic use.

Reported antibiotic use before blood culture was defined as antibiotics received within 72 hours before culture based on patient report and the nurse's review of medications received at the hospital. The name(s) of antibiotics received were determined by patient report or record review. The likely route of antibiotic administration (oral versus intravenous [IV] or intramuscular [IM]) was classified by a Thai physician (S.T.) based on usual hospital practice in Thailand.

Pre-culture antibiotic use was also assessed by measuring serum antimicrobial activity using a serum disc assay. At the time of blood collection, a filter paper disc was coated with 20 μL of patient serum and stored at −70°C. For testing, the filter paper was placed onto a Mueller-Hinton agar plate inoculated with antibiotic-sensitive *Staphylococcus aureus* (ATCC 9144). After 24 hours incubation at 35–37°C, growth inhibition around the disc was considered evidence of serum antimicrobial activity.^4^ An additional measure of antibiotic exposure was created that defined antibiotic use as either reported antibiotic use or serum antimicrobial activity; this group was compared with patients who had no antibiotic use by either measure.

### Blood cultures.

Detailed methods have been described previously.^7^,^8^ Briefly, each blood specimen was divided into a bottle optimized for aerobic growth and into a bottle for enhanced growth of mycobacteria, with the goal of inoculating at least 10 mL from adults and 4 mL from children < 5 years of age into the aerobic bottle and the remainder into the anaerobic bottle. All blood cultures were processed at the two provincial hospital laboratories. Cultures collected at community hospitals were maintained at 15–30°C and transported within 24 hours to the provincial hospitals. Cultures were processed using the BacT/ALERT 3D (bioMerieux, Hazelwood, MO) automated microbial detection system. Media from bottles with evidence of growth as signaled by the automated culture system (i.e., alarm-positive cultures) were sub-cultured using standard methods.^9^ A pre-defined list of common contaminant species was used to determine if a given isolate was a likely pathogen. Confirmatory identification of *Streptococcus pneumoniae* was performed at the reference laboratory of the Thailand Ministry of Public Health and CDC.

### Statistical analysis.

Pearson's χ^2^ tests of association were used to compare clinical characteristics (e.g., fever and respiratory symptoms) by antibiotic use. Agreement between reported antibiotic use and serum disc testing results was assessed using Kappa statistics. The χ^2^ analyses were also used to test associations between antibiotic use and four blood culture endpoints: 1) alarm-positive by automated culture machine; 2) organism isolation; 3) pathogen (i.e., non-contaminant) isolation; and 4) *S. pneumoniae* isolation. For comparison, isolation rates of *Escherichia coli* and *Burkholderia psuedomallei* were examined, expecting the former to be inhibited by antimicrobial activity and the latter to be largely unaffected.

Age group-specific pneumococcal bacteremia incidence rates were calculated by dividing the number of cases by person-years under observation using corresponding population estimates from Thailand's National Economic and Social Development Board.^6^ A Poisson distribution was assumed to calculate 95% confidence intervals (CI). To estimate adjusted incidence rates that accounted for cases missed because of pre-culture antibiotic use, the *S. pneumoniae* isolation rate in patients without antibiotic use was applied to the total number of blood cultures. Differences between observed and adjusted incidence rates were considered statistically significant when the point estimate for the adjusted rate fell outside the range of the 95% CI around the observed rate. Analyses were conducted using SAS version 9.1 (Cary, NC) and SPSS version 15.0 (Chicago, IL).

### Ethical considerations.

These data were collected as part of the public health surveillance activities, which were determined not to require institutional review board review.

## Results

From November 2005 through June 2008, 35,639 blood cultures from 29,883 patients met our criteria for analysis; 6,114 (17%) cultures came from patients who had 2 or more cultures separated by ≥ 7 days. Among patients with > 1 culture, the median time between cultures was 106 days (interquartile range: 33–242 days). Antibiotic use in the 72 hours before blood culture was reported for 9,726 (27%) of 35,639 patient cultures. Similarly, 5,782 (24% of 24,538) blood cultures were from patients with serum antimicrobial activity. Antibiotic use, both reported and by serum antimicrobial activity, differed slightly by province and hospital size (Table 1). Among patients with serum testing, the prevalence of antimicrobial activity increased with age. Serum antimicrobial activity testing was often not performed for patients with small blood volumes collected (e.g., very young children); as a result, patients with serum disc testing results were older on average than those without testing. According to both measures, antibiotic use before blood culture was highest in 2005 and significantly lower each year afterward, (*P* < 0.05).

Detailed information was available for 19,838 patients included in the pneumonia surveillance system. Among these patients, reported antibiotic use prevalence was 28%, and the median age was 29 years. Compared with patients without reported pre-culture antibiotic use, those with antibiotic use were more likely to have fever (38% versus 36%, *P* = 0.03), respiratory symptoms (83% versus 80%, *P* < 0.01), and require supplemental oxygen or intubation (31% versus 29%, *P* < 0.01). The case-fatality proportion was also higher among patients with antibiotic use (4.6% versus 3.5%, *P* < 0.01). Human immunodeficiency virus (HIV) status was available for 5,523 (15.5%) of 35,642 patient cultures; HIV-positive patients had similar rates of pre-culture antibiotic use as HIV-negative patients (28% versus 29%, *P* = 0.33).

Antibiotic names were available for 82% (8,004) of patients with reported antibiotic use. The most commonly reported drugs used were third generation cephalosporins (38%) and penicillin/penicillin derivatives (34%), both in monotherapy or in combination; 1,742 (22%) received ≥ 2 antibiotics. We could determine the likely mode of antibiotic administration for 6,368 (84% of 9,746 with reported antibiotic use); of those, 50% (3,148) had only antibiotics typically administered orally and 50% (3,218) had at least one IM or IV antibiotic.

Correlation between serum antimicrobial activity and reported antibiotic use was moderate. After excluding patients missing antibiotic use data by either measure, 16% of patients were positive for antibiotic by both measures, 62% negative by both, and 23% discordant (Kappa = 0.42). Correlation increased slightly when reported antibiotics were limited to those administered IM or IV (Kappa = 0.48). Correlation was higher for patients aged 5 years and older (0.45) than for children less than 5 years of age (0.27). The major source of discordance, particularly in young children, was negative serum antimicrobial disc testing among patients with reported antibiotic use (Table 2).

Blood culture yields were significantly lower among patients with pre-culture antibiotic use compared with those without antibiotic use (Table 3). This was true when the culture endpoint was alarm positivity, organism isolation, or pathogen isolation, and regardless of whether pre-culture antibiotic use was determined by reported use, serum disc testing, or a combination of the two measures. Overall, *E. coli* was isolated more commonly from those without antibiotic use compared with those with pre-culture antibiotics. In contrast, *B. pseudomallei* yield was higher among patients with pre-culture antibiotic use compared with those without antibiotic use. *Streptococcus pneumoniae* was isolated more often in patients without antibiotics regardless of whether antibiotic use was determined by reported use, serum disc testing, or a combination of the two measures.

When analyses were limited to children < 5 years of age, trends in culture yields for both antibiotic use measures were similar but not statistically significant for pathogen isolation. None of the 554 children < 5 years of age with serum antimicrobial activity had a positive blood culture for *S. pneumoniae*, compared with 12 (0.4%) of 3,291 without serum antimicrobial activity (*P* = 0.09). Unexpectedly, *E. coli* isolation rates were higher among children < 5 years of age with positive serum disc testing (0.9%, *N* = 5; all < 1 year of age) compared with negative serum disc tests (0.2%; *N* = 5).

Culture yield, as measured by alarm positivity, organism isolation, and pathogen isolation, including *S. pneumoniae*, was significantly lower among children < 5 years of age compared with older patients (*P* < 0.05 for all), which may have been caused by lower blood volumes available for culture in children. Less than half (45%) of children < 5 years of age had the target 4 mL available for blood culture and 27.7% had less than 2 mL. *Streptococcus pneumoniae* yield was lowest among children < 5 years of age with < 2 mL collected compared with those with 2–3.99 mL collected and 4 mL or more: 0.1% (*N* = 4), 0.2% (*N* = 7), and 0.3% (*N* = 13), respectively. *Streptococcus pneumoniae* yield within blood volume strata among children < 5 years of age indicated non-significant reductions associated with pre-culture antibiotics, although these analyses are limited by small numbers: no *S. pneumoniae* isolated for both positive and negative serum antimicrobial activity with < 2 mL; 0 (serum antimicrobial activity) versus 0.4% (no serum antimicrobial activity) for 2–3.99 mL; and 0 versus 0.4% for 4 mL or more.

**Figure 1.**
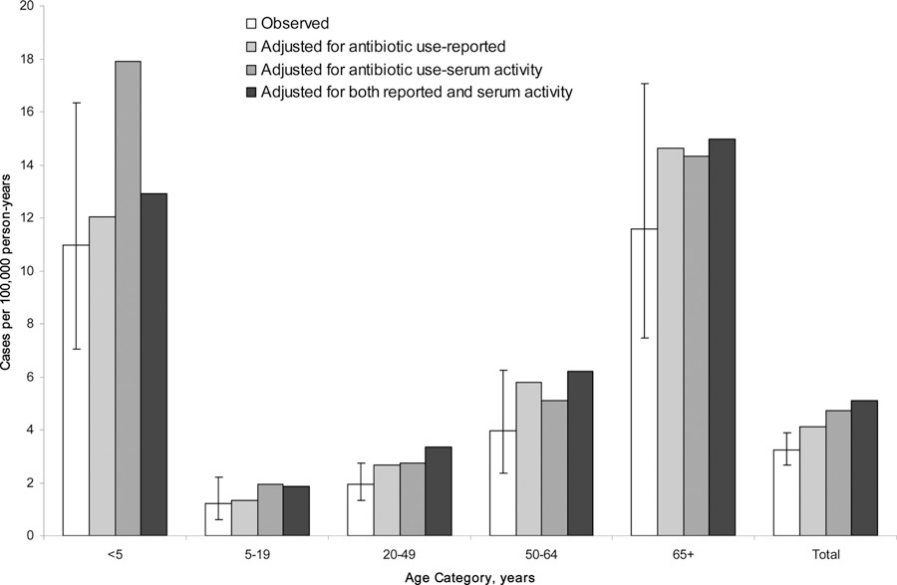
Observed incidence rates of pneumococcal bacteremia requiring hospitalization* and adjusted rates accounting for cases missed because of antibiotic use before culture, as measured by reported antibiotic use, serum antimicrobial activity, and a combination of measures. *This illustration is not intended as a definitive estimate of pneumococcal bacteremia incidence in Sa Kaeo and Nakhon Phanom. A more complete analysis of pneumococcal disease burden in Thailand has been published previously.^8^

Figure 1 illustrates the potential impact of pre-culture antibiotic use on age-specific incidence rates of hospitalized pneumococcal bacteremia. The adjusted incidence rates were significantly greater than the overall observed rate (3.2 per 100,000 person-years; 95% CI = 2.7, 3.9), 60% higher when adjusted for antibiotic use defined as reported use or serum antimicrobial activity (5.1 per 100,000). The adjusted rates within each age category were similar across methods of defining antibiotic exposure, except for young children. Among children < 5 years of age, the adjusted incidence was 63% higher when antibiotic use was defined by serum antimicrobial activity (17.9 versus 11.0 per 100,000 person-years [95% CI for observed incidence = 7.0, 16.3]), 9.6% higher when adjusted for reported antibiotic use, and 18% higher when adjusted for antibiotic exposure defined as either reported use or serum antimicrobial activity. When limited to children < 5 years of age with the targeted 4 mL blood volume, incidence was 40% higher after adjustment for serum antimicrobial activity: 13.0 versus 18.2 per 100,000 person-years.

## Discussion

This investigation used data from active, population-based surveillance for bacteremia and pneumonia to document high rates of pre-culture antibiotic use and show that the frequency of *S. pneumoniae* isolation in patients with reported pre-culture antibiotic use was one-fourth that in those without reported use. Differences in *S. pneumoniae* isolation were even greater when antibiotic use was defined by serum antimicrobial activity or by a combined measure of reported use or serum activity. This lower culture yield significantly reduced the estimated incidence of pneumococcal bacteremia overall and in children < 5 years of age, by 32% and 39%, respectively, when antibiotic use was defined by serum antimicrobial activity.

Our findings were consistent across numerous culture endpoints (alarm positivity, overall isolate yield, and pathogen yield); however, consistent with its fastidious nature, *S. pneumoniae* isolation was affected to a greater degree by pre-culture antibiotic use than pathogen yield overall. These observations are in line with previous studies showing the effects of pre-culture antibiotic use.^2^,^4^,^10^,^11^ To further examine internal consistency, *E. coli* was chosen as a positive control, because it is a frequently cultured organism with known susceptibility to commonly used antibiotics.^12^ As expected, pre-culture antibiotic use significantly reduced *E. coli* yield overall. However, among children < 5 years of age, *E. coli* isolation was higher in patients with pre-culture antibiotic use, an unexpected finding driven by *E. coli* sepsis cases in young infants who were likely treated empirically before culture collection. In contrast, *B. pseudomallei*, an organism endemic in rural Thailand and refractory to one-time *in vivo* antibiotic treatment,^13^ was chosen as a negative control. *B. pseudomallei* recovery was actually higher among those with pre-culture antibiotic use, which may have resulted from patients with more severe disease being both more likely to receive antibiotics early and to have positive cultures. Overall, these findings strengthen the conclusion that differences in culture yield for *S. pneumoniae* by antibiotic use were caused by the antibiotic exposure and not to other factors, such as the amount of specimen collected or HIV status.^4^

Our pre-culture antibiotic use rates (by serum antimicrobial activity) of 24% overall and 14% among children < 5 years of age compare with those of two Kenya studies showing urine antimicrobial activity in ~50% of adults^2^ and serum activity ~10% in children^4^ evaluated for possible sepsis. The high prevalence of pre-culture antibiotic use in our study did not result solely from pre-hospital antibiotic use. The large fraction of IM and IV antibiotics used suggests antibiotics are often administered in the emergency room and/or medical wards before blood cultures are obtained. Pre-culture antibiotic administration in health-care settings may be an underappreciated cause of false-negative blood cultures, especially in areas where access to modern blood culture systems has recently been expanded. From 2005 to 2008 the prevalence of pre-culture antibiotics decreased significantly in our surveillance population (Table 1), possibly related to education emphasizing the importance of culture collection before antibiotic administration. Nonetheless, the magnitude and scope of pre-culture antibiotic use, both in and outside health-care settings, merits further attention in Thailand and in similar settings where antibiotics are available without prescription and bacterial culture-based methods of measuring disease incidence are in place.

This study benefited from the use of two measures of antibiotic use: reported use (based on patient report and nursing records) and serum antimicrobial activity. Although neither measure of antibiotic use is a true gold standard, we found consistent and significant associations across multiple culture endpoints. We found moderate agreement between the two measures with slightly better agreement among patients receiving IM/IV antibiotics and patients 5 years of age and older. Lack of agreement between measurements may have resulted from poor patient recall and/or serum antibiotic levels too low for detection. Testing urine may have been more sensitive than testing serum to detect antimicrobial activity, but urine is difficult to collect on demand at the time of blood culture, especially in children. Poorer correlation in patients < 5 years of age may have been caused by increased misclassification of the reported antibiotic use measure, because children taking antibiotics often miss doses (or do not receive full doses), and non-antimicrobial drugs may get reported as antibiotics. For these reasons, we believe that serum antimicrobial activity serves as the most accurate measure of pre-culture antibiotic use and should be the primary measure used to adjust pneumococcal disease incidence rates for children < 5 years of age (Figure 1).

Our investigation would have benefited from additional data describing the context of antibiotic use (i.e., pharmacy, outpatient clinic, emergency department, or hospital ward). The analysis also relied on blood cultures collected at clinicians' discretion as opposed to standardized enrollment, limiting comparisons to other clinical settings where culture practices may differ. As another limitation, we were unable to stratify the impact of pre-culture antibiotics by degree of anti-pneumococcal activity. All *S. pneumoniae* isolates were sensitive to penicillin,^8^ and too few patients reported taking antibiotics to which non-susceptibility rates were high (e.g., co-trimoxazole), making it difficult to compare the effects of individual antibiotics. Low blood volume also substantially reduced culture yield, and we were unable to fully separate the effects of pre-culture antibiotic use and blood volume in young children because of small numbers. However, there is no reason to think that the microbiological effects of pre-culture antibiotics on culture yield would differ among young children. It should also be noted that our analytic approach assumed that, if not for antibiotics, culture yields would be the same among patients with and without pre-culture antibiotics. In sub-analyses of patients captured by the pneumonia surveillance system, individuals with reported pre-culture antibiotics had similar clinical characteristics to those without antibiotics, supporting this assumption. Finally, the generalizability of our findings may be limited because the impact of pre-culture antibiotic use will vary with local antibiotic use, antibiotic classes used, and pneumococcal susceptibility patterns. However, nonjudicious antibiotic use is common in other parts of Thailand^14^ and Southeast Asia^15^ and other regions of the world^16^ and the impact might be expected to be at least as substantial as what we observed.

The illustration of observed and adjusted pneumococcal bacteremia incidence (Figure 1) is not intended as a definitive estimate of invasive pneumococcal disease incidence in Thailand. A more complete analysis of pneumococcal disease burden in Thailand was published previously^8^ and found rates of bacteremia requiring hospitalization (10.6–28.9 cases per 100,000 in children < 5 years of age) near pre-vaccine rates in the United States and other countries where pneumococcal conjugate vaccine has been introduced. Although our surveillance did not allow us to quantify the effect of pre-culture antibiotic use on overall IPD incidence estimates (both non-hospitalized bacteremia cases as well as IPD cases confirmed by culture of other normally sterile sites are not captured), it is reasonable to conclude that the impact would be similar.

In this study, we described pre-culture antibiotic use in rural Thailand, which has not been detailed for Thailand or other Southeast Asian countries. We showed that pre-culture antibiotics are associated with significantly lower culture yields for *S. pneumoniae*. These findings underscore the limitations of exclusively culture-based methods to estimate *S. pneumoniae* bacteremia and IPD incidence. Our findings are also directly relevant to pneumococcal disease cost estimation and cost-effectiveness analyses of pneumococcal vaccine.^17^,^18^ Alternative methods for detecting *S. pneumoniae*, such as antigen detection^19^ or molecular assays,^20^ should be evaluated in epidemiologic studies and clinical settings for their ability to detect pneumococcal disease in patients pre-treated with antibiotics.

## Figures and Tables

**Table 1:** Antibiotic use before blood culture defined by reported use* and by serum antimicrobial activity; Sa Kaeo and Nakhon Phanom, Thailand, November 2005 to June 2008

	Reported antibiotic use		Serum antimicrobial activity	
	Total	% Yes	Total	% Yes
Total	35639	27.3	24538	23.6
Province				
Sa Kaeo	13070	26.5	10208	24.4
Nakhon Phanom	22569	27.7	14330	23.0
Hospital Type				
Provincial (*N* = 2)	15797	25.5	9208	25.6
Community (*N* = 18)	19842	28.7	15330	22.3
Age in years				
Less than 5	10719	26.3	3845	14.4
5 to 19	3904	29.4	2468	17.5
20 to 49	6939	28.9	6073	24.0
50 to 64	5810	27.5	5069	26.0
65 and greater	8267	26.2	7083	28.5
Median age, years	35.3		49.0	
Year†				
2005	1502	33.9	1176	27.1
2006	12213	30.9	8958	25.6
2007	14757	25.9	9654	21.4
2008	7167	22.8	4750	23.1

* Antibiotics received within 72 hours before culture based on patient report and the nurse's review of hospital medications.

† The year 2005 included only November and December. The year 2008 included January through June.

**Table 2:** Correlation between reported antibiotic use and serum antimicrobial activity by disc testing among patients < 5 years of age*

	Total	Reported antibiotic use†		
		Yes	No	Not sure
Serum disc result	*N* (%)	n (%)	n (%)	n (%)
Positive	554 (14)	348 (31)	195 (8)	11 (5)
Negative	3291 (86)	778 (69)	2281 (92)	232 (96)
Total	3845‡	1126	2476	243

* Kappa = 0.27 (analysis excluded “not sure” reported antibiotic use).

† Antibiotics received within 72 hours before culture based on patient report and the nurse's review of hospital medications.

‡ Serum disc testing was performed for 36% (3,845/10,719) of cultures in children < 5 years of age.

**Table 3:** Blood culture outcomes by reported antibiotic use, serum antimicrobial activity, and a combination of measures, Sa Kaeo and Nakhon Phanom, Thailand, November 2005 to June 2008

	Total	Reported antibiotic use*			Serum antimicrobial activity†			Combined – reported antibiotic use and serum antimicrobial activity‡		
		Yes	No	*P* value	Yes	No	*P* value	Yes by either	No by both	*P* value
	*N* = 35639	*N* = 9726	*N* = 22462		*N* = 5782	*N* = 18756		*N* = 11949	*N* = 13997	
Alarm positive culture§	5393 (15.1)¶	1099 (11.3)	3806 (16.9)	*P* < 0.01	656 (11.3)	3255 (17.4)	*P* < 0.01	1404 (11.7)	2577 (18.4)	*P* < 0.01
Organism isolation	4729 (13.3)	910 (9.4)	3387 (15.1)	*P* < 0.01	521 (9.0)	2857 (15.2)	*P* < 0.01	1164 (9.7)	2282 (16.3)	*P* < 0.01
Pathogen isolation‖	2419 (6.8)	393 (4.0)	1813 (8.1)	*P* < 0.01	252 (4.4)	1650 (8.8)	*P* < 0.01	529 (4.4)	1343 (9.6)	*P* < 0.01
*Escherichia coli*	843 (2.4)	92 (0.95)	675 (3.0)	*P* < 0.01	81 (1.4)	633 (3.4)	*P* < 0.01	139 (1.3)	545 (3.9)	*P* < 0.01
*Burkholderia pseudomallei*	321 (0.90)	105 (1.1)	186 (0.83)	*P* = 0.03	92 (1.6)	173 (0.93)	*P* < 0.01	148 (1.2)	117 (0.8)	*P* < 0.01
*Streptococcus pneumoniae*	109 (0.31)	9 (0.09)	83 (0.37)	*P* < 0.01	3 (0.05)	84 (0.45)	*P* < 0.01	12 (0.10)	68 (0.49)	*P* < 0.01

* Antibiotics received within 72 hours before culture based on patient report and the nurse's review of hospital medications. *P* values reflect row-wise comparisons of “Yes” vs. “No” reported antibiotic use; “Not Sure” excluded, *N* = 3451.

† *P* values reflect row-wise comparisons of “Yes” vs. “No” serum antimicrobial activity; “Missing” excluded, *N* = 11101.

‡ *P* values reflect row-wise comparisons of “Yes by either” vs. “No by both,” “Unknown” excluded, (*N* = 9693). The “Unknown” category includes: 1) patients with “no” reported antibiotic use and “missing” serum antimicrobial activity, 2) patients with “unsure” reported antibiotic use and “negative” serum antimicrobial activity, and 3) “unsure” reported antibiotic use and “missing” serum antimicrobial activity.

§ BacT/ALERT machine alarmed signaling a positive culture.

¶ Parentheses show column percentages.

‖ Non-contaminant species isolated from subculture.
